# Sex‐dependent alterations of dopamine receptor and glucose transporter density in rat hypothalamus under long‐term clozapine and haloperidol medication

**DOI:** 10.1002/brb3.1694

**Published:** 2020-06-11

**Authors:** Marie‐Luise Bouvier, Karin Fehsel, Andrea Schmitt, Eva Meisenzahl‐Lechner, Wolfgang Gaebel, Martina von Wilmsdorff

**Affiliations:** ^1^ Department of Psychiatry and Psychotherapy Medical Faculty Heinrich‐Heine‐University Düsseldorf Germany; ^2^ Department of Psychiatry and Psychotherapy University Hospital Ludwig‐Maximilians University Munich München Germany; ^3^ Laboratory of Neuroscience (LIM27) Institute of Psychiatry University of Sao Paulo São Paulo Brazil

**Keywords:** clozapine, dopamine receptors, glucose transporters, haloperidol, sex differences

## Abstract

**Objective:**

Sex‐dependent disturbances of peripheral glucose metabolism are known complications of antipsychotic drug treatment. The influence of long‐term clozapine and haloperidol medication on hypothalamus, maintaining aspects of internal body homeostasis, has not yet been completely clarified.

**Methods:**

After puberty, male and female Sprague Dawley rats were fed orally with ground pellets containing haloperidol (1 mg/kgBW/day) or clozapine (20 mg/kgBW/day) for 12 weeks. The hypothalamic protein expression of dopamine receptors D2R and D4R, melanocortin receptor MC4R, and glucose transporters Glut1 and Glut3 was examined. Glucose, glycogen, lactate, and pyruvate levels were determined, also malondialdehyde equivalents as markers of oxidative stress.

**Results:**

D2R expression was increased in the male haloperidol and clozapine group but decreased in females medicated with clozapine. D4R expression was upregulated under clozapine medication. While females showed increased Glut1, Glut3 was elevated in both male and female clozapine‐medicated animals. We found no changes of hypothalamic malondialdehyde, glycogen, and MC4R. Hypothalamic lactate was elevated in the female clozapine group.

**Conclusion:**

Clozapine sex‐dependently affects the expression of D2R, Glut1, and Glut3. The upregulation of the glucose transporters indicates glucose deprivation in the endothelial cells and consequently in astrocytes and neurons. Increased hypothalamic lactate in females under clozapine points to enhanced glycolysis with a higher glucose demand to produce the required energy. Haloperidol did not change the expression of the glucose transporters and upregulated D2R only in males.

## Graphical abstract

We have studied the long‐term effect of clozapine or haloperidol on hypothalamic glucose metabolism in adult male and female Sprague Dawley rats. We have found sex‐dependent changes in dopamine receptors and glucose transporters indicating a higher glucose demand under clozapine.

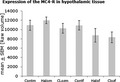

## 1 INTRODUCTION

Schizophrenia represents a complex disease. Various lines of evidence suggest an underlying impairment of cerebral energy supply (Pillinger et al., 2017; Pinacho et al., 2016). Successful symptomatic treatment of schizophrenia with the atypical antipsychotic drug (APD) clozapine and the typical APD haloperidol can produce severe metabolic disturbances, but the underlying mechanisms remain unclear. In previous studies (von Wilmsdorff et al., 2013; von Wilmsdorff et al., 2014) on healthy Sprague Dawley rats, we demonstrated more severe sex‐dependent peripheral changes of glucose metabolism under clozapine than under haloperidol. Clozapine increased body weight, fat and liver mass in males, whereas increased serum glucose in females was associated with decreased hepatic glycogen levels. Food and water intake per week was decreased under haloperidol medication in both sexes but unchanged under clozapine treatment. Food intake related to 1 kg body weight was decreased in rats medicated with haloperidol and in males under clozapine treatment, but not in females.

The hypothalamus as the most crucial control center of the autonomic nervous system is considered an important target for APDs. It is responsible for information exchange between brain and periphery, regulating food intake, energy consumption, and metabolic processes in combination with the hypothalamic melanocortin system, which plays a key role in these regulations (Romanova et al., 2018). There is evidence that the neuronal dopaminergic system participates not only in the etiopathology of schizophrenia but also in the effectiveness of APDs (Brisch et al., 2014). The therapeutic efficacy of both first‐ and second‐generation antipsychotic medication is mechanistically linked to blockade of the dopamine receptor 2 (D2R) (Nash, 2017). Haloperidol exhibits high affinity to D2R with slow receptor dissociation kinetics, remaining attached for days, in contrast to clozapine which is released from D2R within 12–20 hr (Seeman, 2011). It has been proposed that a much faster dissociation from the D2R distinguishes atypical from typical aantipsychotics. However, recent research findings cast doubt on the proposal that typical and atypical antipsychotics can be primarily distinguished by their D2R binding kinetics (Sahlholm et al., 2016). Furthermore, G protein‐coupled receptors like dopamine receptor 2 and dopamine receptor 4 are able to interact with other receptors by forming constitutive heterodimers, and antipsychotic drugs like clozapine enhance this connection, thus adding an additional layer of pharmacological complexity (Lukasiewicz, Blasiak, & Szafran‐Pilch, 2016; Woods, 2010).

Inside the brain, dopamine and its receptors are involved in many neurological processes such as cognition, memory, learning, motor control, modulation of neuroendocrine signaling, and regulation of homeostasis (Beaulieu, Del'Guidice, Sotnikova, Lemasson, & Gainetdinov, 2011). Dopamine regulates oxidative phosphorylation (Barros‐Minones, Goni‐Allo, Suquia, Beitia, & Aguirre, 2015) as well as glucose uptake by the PI3K/Akt pathway (Khorami, Movahedi, Huzwah, & Sokhini, 2015), and dopamine receptors modulate the expression of insulin receptor and glucose transporter 3 (Glut3) in the rat brain (Anitha & Abraham, 2012). Dopaminergic signaling mediates the acute anorectic effect of melanocortin stimulation and contributes to food intake and body weight regulation (Billes, Simonds, & Cowley, 2012). Impairments of this system lead to metabolic disorders like obesity or metabolic syndrome. Significant colocalization of the melanocortin receptor MC4R and D2R was found in the hypothalamic arcuate nucleus (ARC), pointing to tight functional interactions of the two systems (Derkach, Romanova, & Shpakov, 2018).

Apart from the largely empirical dopamine theory, impaired neuronal glucose uptake is discussed in the pathogenesis of schizophrenia (McDermott & de Silva, 2005). Glucose transporters are membrane‐bound protein transporters catalyzing the transport of glucose or fructose through the cell membrane. Glut1 and Glut3 are expressed particularly in the central nervous system and erythrocytes. Both transporters are insulin‐independent, and the concentration gradient of glucose provides the energy needed for transport. Glut1 transports glucose across the endothelial cells of the blood–brain barrier (BBB). Then, glucose passes by the neuron‐specific Glut3, the most abundant glucose transporter in brain, through the cell membrane into the neurons (Mergenthaler, Lindauer, Dienel, & Meisel, 2013). Clozapine probably participates in the apoptosis of the human endothelial cells of the BBB with an impairment of barrier functionality (Elmorsy, Elzalabany, Elsheikha, & Smith, 2014) but seems to protect dopaminergic neurons by inhibiting microglial overactivation, ROS production, and oxidative stress (Hu et al., 2012).

In our recent studies (von Wilmsdorff et al., 2013; von Wilmsdorff et al., 2014), we have examined peripheral metabolic effects of clozapine and haloperidol in blood and liver, although the central mechanisms remained unclear. Therefore, the purpose of this explorative study was to examine basic metabolic changes in the hypothalamus, including the expression of receptors and transporters, involved in the hypothalamic glucose metabolism in male and female rats, fed orally with the atypical clozapine or the typical haloperidol over a chronic period of 12 weeks.

## 2 METHODS

### 2.1 Animals, antipsychotic administration, and tissue collection

The detailed experimental conditions of the rat feeding study and the results of blood and liver examinations have been described earlier (von Wilmsdorff et al., 2013; von Wilmsdorff et al., 2014). All experiments were carried out in accordance with the laws of the local authorities for animal experiments approved by the Landesamt für Natur, Umwelt‐ und Verbraucherschutz NRW, Recklinghausen (Reference number 9.93.2.10.34.07.227). In brief, haloperidol (1 mg/kg body weight [BW]/day) or clozapine (20 mg/BW/day) was fed orally in ground pellets (Altromin maintenance diet with 19% crude protein, 4% crude fat, additionally 15% fat and cereals as carbohydrate source) to groups of 10 male and female Sprague Dawley rats (Taconic) beginning at postnatal day (PD) 85 over a 12‐week period. All animals were individually housed. The control group was fed with ground pellets only. Twelve hours after food removal in the dark cycle, the main period of food intake and motor activity of rats, the animals were anesthetized by pentobarbital (Narcoren, Merial) on PD169. Following blood withdrawal, the brains were removed and frozen immediately at −80°C by 2‐methylbutane, cooled down by liquid nitrogen, and stored at −80°C. Frozen frontal sections (60 µm) were cut on a cryostat (CM3000; Jung) and the hypothalamic regions around the ventricle with a diameter of 3 were excised by sterile biopsy punches (kai Europe GmbH), according to a rat brain atlas (Paxinos & Watson, 1999).

### 2.2 Metabolic parameters

All metabolic assays were carried out with the supernatant of tissue preparation (see Western blot analysis). Total glucose levels in brain tissue and serum were determined spectrophotometrically at 530 nm by a glucose assay kit (Sigma‐Aldrich) following the manufacturer's instructions. Lactate and pyruvate levels were also detected colorimetrically at 530 or 570 nm, respectively, by using a lactate (Labor + Technik, Eberhard Lehmann GmbH) or pyruvate (BioAssay Systems) assay kit.

Albumin levels were determined colorimetrically at 630 nm by the bromocresol green reaction (Labor + Technik, Eberhard Lehmann GmbH). Total protein content of the cytosolic and membrane fraction was measured using the DC Protein Assay from Bio Rad.

### 2.3 Lipid peroxidation

Malondialdehyde (MDA), the degradation product of polyunsaturated lipids, is used as a biomarker to measure the level of oxidative stress. In the present study, we used the assay of Ohkawa, Ohishi, and Yagi (1979) in animal tissues by thiobarbituric acid reaction to determine the thiobarbituric acid reactive substances (TBARS). One hundred microliter of hypothalamic tissue lysate were used. Spectral absorption was measured at 532 nm, and malondialdehyde equivalents were calculated in µmol/ml by a standard curve.

### 2.4 Glycogen

To assess glycogen levels, 50 µl of hypothalamic tissue lysate were added to 100 µl HClO_4_ (ice cold, 6%), heated in a water bath at 70°C for 10 min and centrifuged at 1,000 *g* for 10 min at 4°C. Two hundred microliter methanol were added to 100 µl of the supernatant, heated in a water bath at 37°C for 30 min and centrifuged at 13,000 *g* for 10 min at room temperature. The pellet was dissolved in 100 µl aqua dest and 200 µl anthrone reagent (0.2% anthrone [Sigma‐Aldrich] freshly dissolved in concentrated H_2_SO_4_) was added to the solution. The extinction of the solution could be measured at 630 nm, and the levels of glycogen were calculated in ng/ml of hypothalamic tissue using a standard curve.

### 2.5 Western blot analysis of dopamine D2 and D4 receptor, glucose transporter 1 and 3, and melanocortin 4 receptor

Hypothalamic tissue (0.1 g) was washed three times with KCl buffer (1.15%) and then homogenized in 450 µl 1.15% KCl, gently shaken on ice for 30 min and centrifuged at 13,000 *g* for 10 min at 4°C. The cytosolic supernatant was stored at −80°C for the determination of glucose, albumin, lactate, pyruvate, and the malondialdehyde test. The pellet was suspended in 200 µl RIPA buffer with 4% Triton‐X100 (Sigma‐Aldrich), 1 mM PMSF (Sigma‐Aldrich), and Complete Protease Inhibitor Cocktail Tablets (Roche Diagnostics) overnight at 4°C with gentle shaking (Wang et al., 2010). The lysate was centrifuged at 13,000 *g* for 10 min at 4°C. Total protein content of the supernatant was measured using the DC Protein Assay from Bio Rad. Twenty‐five microgram protein from each animal was separated on NuPAGE™ 4%–12% Bis‐Tris gels (Thermo Fisher scientific) and blotted onto Invitrolon membranes (Thermo Fisher scientific). Membranes were then blocked with Roti Block (Carl Roth) in TBS‐T (1:10) at 4°C for 4 hr. The blocked PVDF membranes were incubated overnight at 4°C either with rabbit polyclonal D2R antibody (1:1,000; Santa Cruz Biotechnology, RRID: sc‐9113) or rabbit polyclonal D4R antibody (1:1,000; Abcam, RRID: ab20424) antibody in TBS‐T and then probed at room temperature for 1 hr with the HRP‐conjugated secondary antibody (1:10,000; Abcam; RRID: ab97051, m). Resulting autoradiographs were quantified by densitometry using Gene Snap and Gene Tools software (Syngene, Synoptics Ltd). The membranes were routinely stained with Ponceau S before immunostaining, in order to confirm correct samples loading and the transfer of equivalent amounts of protein (Romero‐Calvo et al., 2010).

Expression of MC4R, Glut1, and Glut3 was determined on the same membranes after stripping with Roti‐Free stripping buffer 2.0 (Carl Roth). The overnight incubation was done with mouse monoclonal MC4‐R antibody (1:400; Santa Cruz Biotechnology, RRID: sc‐55567), rabbit polyclonal Glut1 antibody (1:500; Santa Cruz Biotechnology, RRID: sc‐7903), and Glut3 antibody (1:500, Santa Cruz Biotechnology, RRID: sc‐30107).

### 2.6 Statistical analysis

Statistical analysis was performed using the IBM SPSS version 22.0 for windows. All data were presented as mean ± *SEM*, and all tests were two‐tailed. Our results show a normal distribution of the data by Kolmogorov–Smirnov test on normality allowing analysis by parametric tests.

The data were examined by two‐way ANOVA with the between‐subject factors MEDICATION and SEX. In case of significant group effects, appropriate post hoc tests were carried out between the groups. Sex‐dependent effects were examined by *t* tests for independent samples. Due to the small number of test animals and the explorative character of the study, *p*‐values ≤ .05 were considered as statistically significant and multiple testing was presented without α‐adjustment.

Based on the assumption that D2R is collocated with MC4R, which modulates food intake by insulin secretion, a bivariate correlation procedure with Spearman‐Rho coefficient was carried out to test the relationship between MC4R, food intake, serum insulin, and ghrelin level with *p* ≤ .05 as significant. Given that lactate and pyruvate levels depend on the glycolytic state of the cells, the correlation of glucose, pyruvate, and lactate was also tested.

## 3 RESULTS

### 3.1 Basic metabolic parameters

Serum levels of haloperidol, clozapine, and its metabolite N‐desmethylclozapine, body weight, motor activity, fat tissue, food and water intake (raw data and calculated on 1 kg body weight), and metabolic parameters have been mentioned previously (von Wilmsdorff et al., 2013) (Table 1). In short, male and female rats treated with haloperidol gained less weight than the control groups, associated with reduced food intake. Male rats under clozapine medication showed significantly increased body weight, fat tissue, and liver mass, although their food intake was reduced. Increased fasting glucose in serum and decreased glycogen in liver were found in female rats treated with clozapine (von Wilmsdorff et al., 2014).

**TABLE 1:** Mean and standard error of hypothalamic total protein, albumin, malondialdehyde, total glucose, glycogen, lactate, pyruvate levels, and lactate/pyruvate ratio of male and female Sprague Dawley rats

	Male control	Male halo	Male cloz	Female control	Female halo	Female cloz
Protein level membrane						
Hypothalamus (mg/ml)	9.08 ± 0.23	8.67 ± 0.14	8.74 ± 0.34	9.10 ± 0.31	8.86 ± 0.22	8.89 ± 0.33
Protein level cytosol						
Hypothalamus (mg/ml)	9.17 ± 0.40	10.00 ± 0.35	9.11 ± 0.40	8.76 ± 0.33	8.49 ± 0.44	9.20 ± 0.46
Albumin						
Hypothalamus (mg/ml)	9.14 ± 0.81	8.99 ± 0.59	7.3 ± 0.53	7.22 ± 0.60	7.39 ± 0.51	8.00 ± 0.54
Oxidative stress						
Hypothalamus (MDAµmol/ml)	0.037 ± 0.007	0.029 ± 0.006	0.025 ± 0.006	0.034 ± 0.01	0.032 ± 0.01	0.034 ± 0.007
Fasting glucose						
Serum (µg/ml)	47.46 ± 5.44	64.05 ± 4.58	57.22 ± 3.81	34.37 ± 1.59	40.33 ± 5.45	45.42 ± 2.99*
Total glucose						
Hypothalamus (µg/ml)	8.70 ± 2.1	13.59 ± 3.2	7.89 ± 1.5	7.02 ± 1.2	8.85 ± 1.5	9.41 ± 1.9
Glycogen						
Hypothalamus (µg/ml)	4.87 ± 0.52	4.38 ± 0.53	3.98 ± 0.75	5.24 ± 0.59	5.16 ± 0.91	5.41 ± 0.64
Lactate						
Hypothalamus (mmol/L)	2.06 ± 0.072	2.14 ± 0.076	2.25 ± 0.085	2.05 ± 0.059	2.21 ± 0.072	2.31 ± 0.095*
Pyruvate						
Hypothalamus (µmol/L)	64.64 ± 11.5	58.14 ± 7.1	47.71 ± 10.3	50.07 ± 12.02	41.93 ± 5.0	63.64 ± 13.8
Lactate/pyruvate ratio						
Hypothalamus	40.95 ± 6.2	41.42 ± 4.8	70.04 ± 14.7	68.67 ± 16.9	59.23 ± 6.5	58.33 ± 17.3

Note

cloz, clozapine medicated; halo, haloperidol medicated; with *n*_group_ = 10.

* *p* < .05 and **p < .001 as significant.

We found no differences of the hypothalamic total cytosol and membrane protein amount and albumin (Table 1). Although malondialdehyde as a marker of oxidative stress was significantly increased in liver tissue of clozapine‐ and haloperidol‐medicated rats (von Wilmsdorff et al., 2014), we found no evidence for oxidative stress manifested in increased lipid peroxidation in the hypothalamus after antipsychotic medication (Table 1). Hypothalamic glycogen content was not changed between the groups in terms of medication or sex (Table 1).

Statistical analysis showed no significant effect of the two antipsychotic drugs on hypothalamic total glucose (Table 1). Serum fasting glucose levels differed significantly for MEDICATION [*F*(2,53) = 7.04; *p* = .002] and SEX [*F*(1,53) = 21.8; *p* = .000021]. Post hoc tests showed significant differences between female control and clozapine‐medicated rats (*p*_femalecontrol:femaleclozapine_ = .00012). Male control and clozapine‐medicated animals differed significantly from females (*p*_controlmale:female_ = .002; *p*_clozapinemale:female_ = .028). Hypothalamic pyruvate levels did not differ significantly between the groups (Table 1), but we found positive correlations between hypothalamic lactate and pyruvate values in the male (*ρ* = .637; *p* = .048) and the female (*ρ* = .647; *p* = .043) haloperidol‐medicated group. The hypothalamic lactate levels (Table 1) differed significantly for MEDICATION [*F*(2,54) = 3.99; *p* = .024] with significant differences between female control and clozapine‐medicated rats (*p*_femalecontrol:femaleclozapine_ = .048).

### 3.2 Receptors and transporters

#### 3.2.1 Dopamine receptors

**FIGURE 1:**
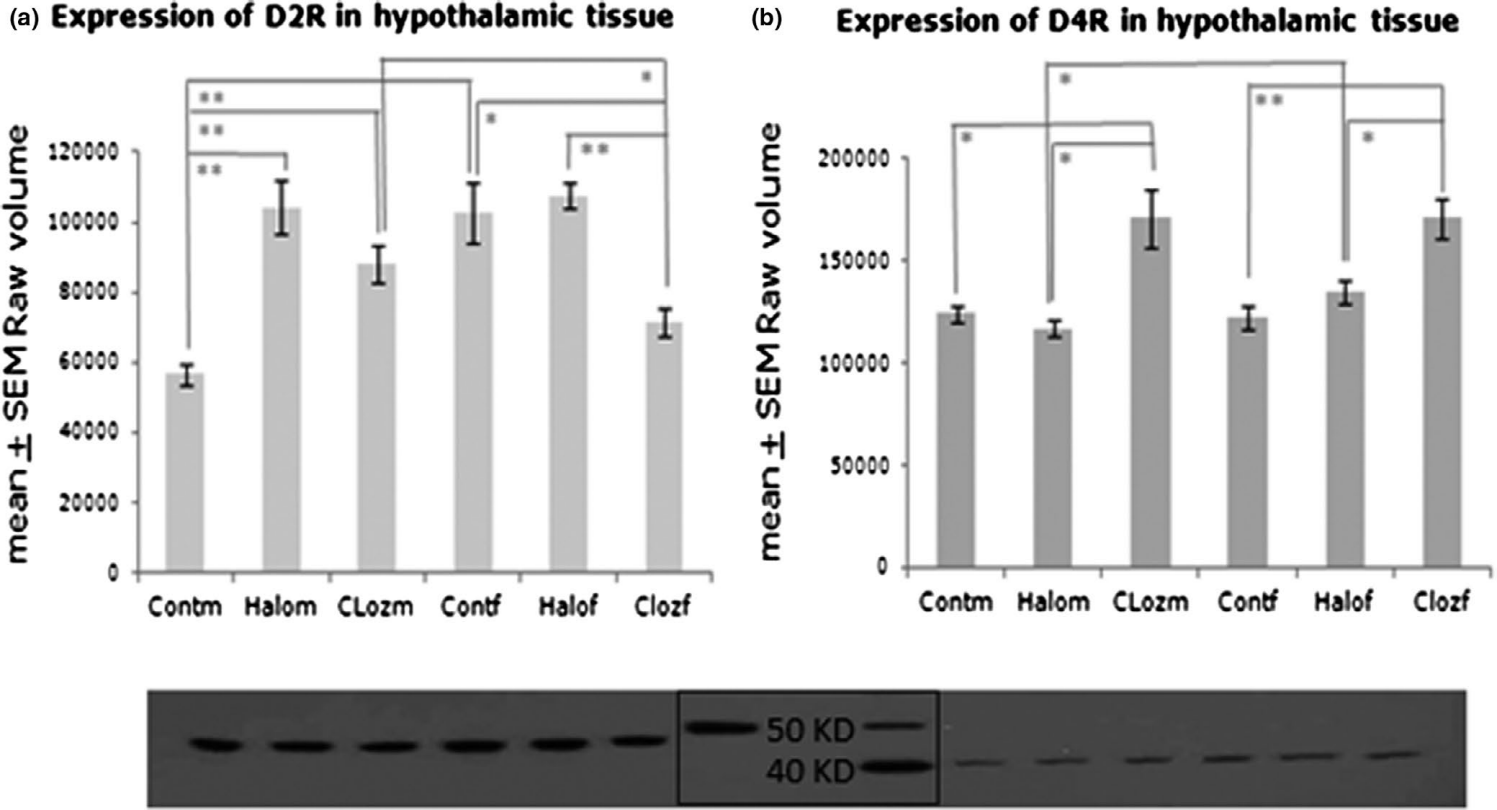
Expression of the dopamine 2 (D2R) and dopamine 4 (D4R) receptor in hypothalamic membrane fraction after 12‐week medication of clozapine or haloperidol in male and female Sprague Dawley rats (Cloz, clozapine medicated; Cont, control; f, female; Halo, haloperidol medicated; KD, Kilo Dalton; m, male). *n*_group_ = 10 with *p ≤ .05 and **p < .001. The autoradiographs show the dopamine receptor expression of pooled values of each treatment group

We found differences for MEDICATION [*F*(2,49) = 15.64; *p* = .000006], SEX [*F*(1,49) = 6.09; *p* = .017], and MEDICATION × SEX [*F*(2,49) = 16.79; *p* = .000003] in the expression of the hypothalamic D2R and differences for MEDICATION [*F*(2,49) = 22.99; *p* < .000001] of the D4R (Figure 1).

Under haloperidol medication, the D2R was significantly increased in males (*p* = .00045), but not in females. Under clozapine medication, D2R was also significantly increased in male rats (*p* = .00056), whereas it was significantly decreased in females (*p* = .0026). Female haloperidol‐treated animals differed significantly from the female clozapine group (*p* = .00029). The expression of D2R differed significantly between males and females of the control (*p* = .001) and clozapine group (*p* = .021).

Male clozapine‐medicated rats showed increased expression of D4R in comparison with the control group (*p* = .039) and to the male haloperidol group (*p* = .018). The D4R was also significantly increased in female clozapine‐medicated animals in comparison with female controls (*p* = .0002) and to female haloperidol‐medicated rats (*p* = .005). Males under haloperidol medication differed from females in the expression of D4R (*p* = .018).

#### 3.2.2 Glut1 and Glut3

**FIGURE 2:**
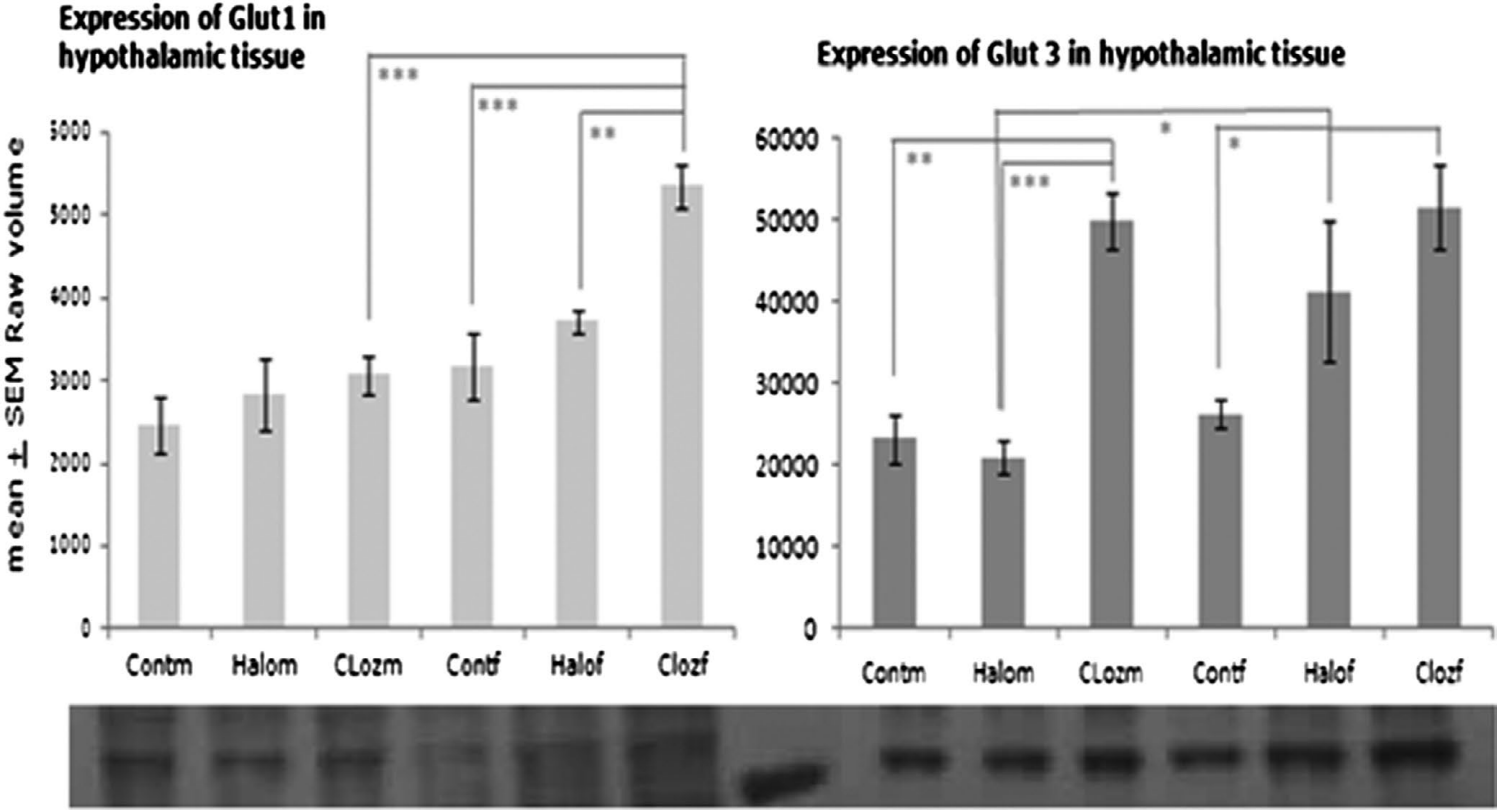
Expression of the glucose transporters Glut1 and Glut3 in hypothalamic membrane fraction after 12‐week medication of clozapine or haloperidol in male and female Sprague Dawley rats (Cloz, clozapine medicated; Cont, control; f, female; Halo, haloperidol medicated; m, male), *n*_group_ = 10 for Glut1 and *n*_group_ = 5 for Glut3 with *p ≤ .05, **p < .001 and ***p < .0001. The autoradiographs show the glucose transporter expression of pooled probes of each treatment group

We found differences in hypothalamic Glut1 expression for MEDICATION [*F*(2,50) = 0.59; *p* = .00015], SEX [*F*(1,50) = 4.05; *p* = .000005], and MEDICATION × SEX [*F*(2,50) = 4.05; *p* = .023] (Figure 2). Female clozapine‐medicated rats showed a higher expression of Glut1 compared to the female controls (*p* = .00005) and the female haloperidol‐medicated animals (*p* = .001). Male and female clozapine‐treated rats differed significantly from each other (*p* = .000005).

The expression of Glut3 differed for MEDICATION [*F*(1,24) = 17.22; *p* = .000023] and SEX [*F*(1,24) = 4.91; *p* = .037]. Male controls and haloperidol‐medicated rats differed significantly from the male clozapine group (*p*_control:clozapine_ = .00011 and *p*_haloperido:clozapine_ = .000048). Only in the haloperidol‐medicated rats, males differed significantly from females (*p* = .050). Increased expression of Glut3 was found in female clozapine‐treated rats in comparison with controls (*p* = .032).

#### 3.2.3 MC4R

**FIGURE 3:**
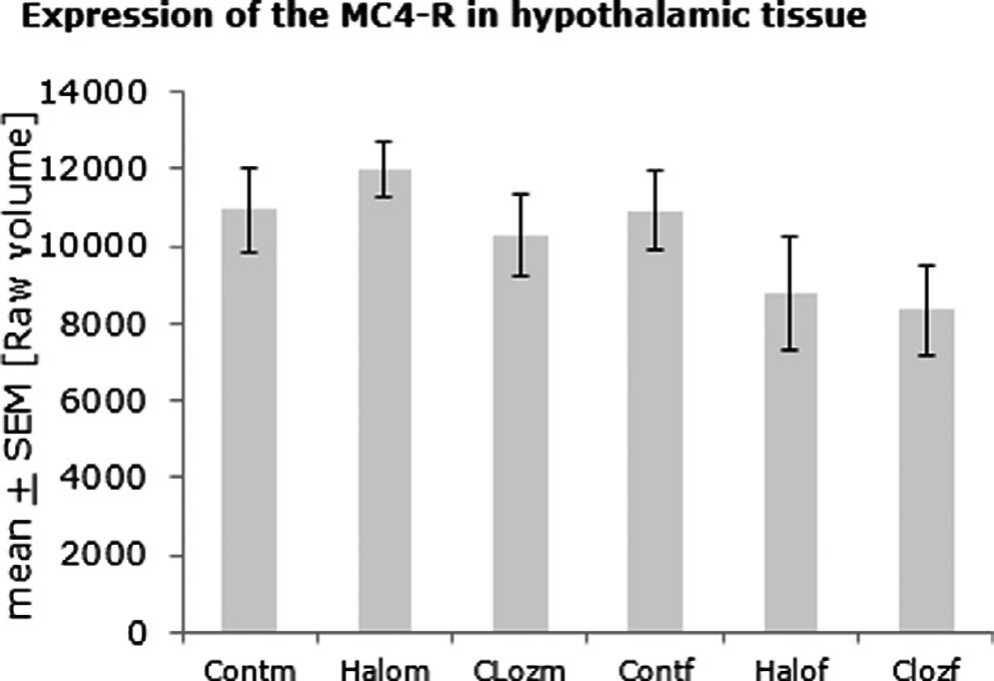
Expression of the melanocortin 4 receptor (MC4R) in hypothalamic membrane fraction after 12‐week medication of clozapine or haloperidol in male and female Sprague Dawley rats (Cloz, clozapine medicated; Cont, control; f, female; Halo, haloperidol medicated; m, male). *n*_group_ = 10

We did not find significant differences in the expression of MC4R (Figure 3 ). Negative correlations were found for the serum insulin level and the expression of hypothalamic MC4R for male haloperidol (*ρ* = −.790 with *p* = .02) and male clozapine‐treated animals (*ρ* = −.675 with *p* = .032). Ghrelin serum levels and hypothalamic MC4R are negatively correlated in male haloperidol‐medicated animals (*ρ* = −.778 with *p* = .023).

## 4 DISCUSSION

Based on the above‐mentioned analysis of fundamental peripheral metabolic parameters of clozapine and haloperidol medication over 12 weeks like body weight gain, fat and liver mass, food and water intake as well as intrinsic factors as fasting glucose, glycogen, leptin, insulin, ghrelin, oxidative stress, triglycerides, and cholesterol (von Wilmsdorff et al., 2013; von Wilmsdorff et al., 2014), we have now studied basic effects of the two APDs on brain glucose metabolism of the same rat trial. We have found that hypothalamic expression of D2R and D4R varies between clozapine and haloperidol, compared to controls, but also between males and females. D2R was upregulated in male rats under haloperidol and to a lesser extent under clozapine medication and was downregulated in female rats under clozapine, whereas only clozapine increases D4R expression in male and female rats. Receptor antagonism was counter regulated by increased receptor expression and the observed alterations reflect the high affinity of haloperidol to D2R, whereas clozapine is able to bind both receptors, however with a hypothesized higher affinity to D4R (Durcan, Rigdon, Norman, & Morgan, 1995). Medication of antipsychotic drugs, which block dopamine receptors, may cause hyperinsulinemia, increased weight gain and glucose intolerance (Lopez Vicchi et al., 2016). D2R is involved not only in schizophrenia, but also in elevated levels of blood glucose in schizophrenic patients (Lawford et al., 2016). Glucose modulates striatal dopamine in mice, whereas striatal D2R regulates peripheral glucose levels and is implicated in glucose‐related metabolic disorders (Michaelides et al., 2017). Mice lacking D2R are glucose intolerant and have abnormal insulin secretion (Lopez Vicchi et al., 2016). Activation of D4R in the hypothalamic paraventricular nucleus of male Wistar rats stimulates food intake by inhibiting satiety (Tejas‐Juarez et al., 2014) in contrast to our results on SD rats, which shows no effect on food intake after clozapine‐induced D4R enhancement. Differences in body weight gain, food intake, and endocrine metabolism are well known in different rat strains (Kühn, Bellon, Huybrechts, & Heyns, 1983; Swerlow, Varty, & Geyer, 1998). Sex‐dependent differences of D2R expression are found in the control and clozapine group (Figure 1), suggesting that the opposite downregulation of D2R expression in female clozapine‐medicated rats might partially be responsible for sex‐dependent metabolic changes and not the binding on D4R. Recent studies (Georgiou et al., 2018; Okita et al., 2016) found sex‐dependent receptor diversity in dopaminergic neuronal systems, which is in line with our results.

D2R activation may protect oligodendrocytes from neurotoxicity induced by oxidative stress, hypoxia, or glucose deprivation (Rosin, Colombo, Calver, Bates, & Skaper, 2005). In contrast to liver tissue (von Wilmsdorff et al., 2014), we find no elevation of hypothalamic malondialdehyde, thereby excluding elevated membrane lipid peroxidation by haloperidol or clozapine (Table 1). However, the experimental results are contradictory and haloperidol or clozapine have been shown to be neuroprotective against oxidative stress, but also cytotoxic to neurons. Andreazza et al. (2015) could show that a 28‐day administration of haloperidol in male SD rats slightly increased lipid peroxidation in gray matter, whereas clozapine increased MDA levels only in liver in accordance with our results. In male Wistar rats, chronic haloperidol treatment caused membrane lipid degradation in rat brain after 45 and 90 days, whereas clozapine did not (Parikh, Khan, & Mahadik, 2003). There is evidence that oxidative damage, mediated by antipsychotic drugs, depends on the brain region. Haloperidol and clozapine could induce oxidative stress in the striatum (Polydoro et al., 2004). In the hippocampus, protein carbonyls were increased by clozapine and haloperidol, whereas TBARS levels were not altered in the cortex (Reinke et al., 2004). Further investigations are needed to clarify, if the hypothalamus is one of the brain regions, which are protected from oxidative stress and, if so, how this protection works.

Mitochondrial damage, lack of oxygen, or hypoglycemia reduces the ATP production via restricted oxidative phosphorylation (OXPHOS) and thereby decreases the reactive oxygen species (ROS) production (Rosin et al., 2005). However, more glucose is needed to maintain ATP demand by enhanced glycolysis, thereby accumulating lactate. Contreras‐Shannon et al. (2013) reported mitochondrial damage induced by clozapine, but not by haloperidol. Indeed, increased hypothalamic lactate levels were seen in clozapine‐medicated rats, which was significant for females (Table 1). In cultured oligodendrocytes, clozapine improves glucose uptake, production and release of lactate, referring to enhanced glycolysis, whereas haloperidol leads to higher extracellular levels of glucose and lower levels of lactate, suggesting reduced glycolysis (Steiner et al., 2014). In contrast to studies on cultured cells, we cannot differ between extra‐ and intracellular levels of glucose or lactate, but we also hypothesize that clozapine enhances glycolysis to provide the required ATP demand in rat hypothalamus. In contrast to liver, where glycogen deposits are apparently transformed to serum glucose (von Wilmsdorff et al., 2014), we did not find significant differences of hypothalamic glycogen with a trend to reduced glycogen levels in clozapine‐medicated rats and increased levels in females under clozapine medication (Table 1). In male Sprague Dawley rats, Duarte, Morgenthaler, and Gruetter (2017) could show that glycogen levels were unaltered in the hypothalamus after hypoglycemia. In the ventromedial hypothalamic nucleus, estrogen receptor‐alpha and estrogen receptor‐beta (ER‐α and ER‐β) enhance astrocyte AMP‐activated protein kinase (AMPK) and glycogen synthase expression and inhibit glycogen phosphorylase in hypoglycemic female SD rats, while ER‐ß suppresses the same proteins in male SD rats (Hasan Mahmood et al., 2018), thus explaining the trend to decreased glycogen levels in males and increased levels in females.

Acute administration of clozapine seems to effectively inhibit glucose transport and to produce hyperglycemia in mice, whereas haloperidol shows only marginal effects (Dwyer & Donohoe, 2003). In contrast, clozapine improved glucose uptake in cultured oligodendrocytes, whereas haloperidol led to higher extracellular levels of glucose (Steiner et al., 2014). Increased levels of glucose transporter Glut1 and mainly Glut3 are signs of cellular hypoglycemia in the brain, indicating that a long‐term local upregulation of Gluts is a response to increased local cerebral glucose utilization (Duelli & Kuschinsky, 2001). In our study, Glut1 and Glut3 are significantly upregulated in the female clozapine group and to a lesser extent in the female haloperidol group (Figure 2), indicating a disturbed glucose supply already in the endothelial cells of the BBB or astrocytes, whereas in male clozapine‐medicated rats only the expression of Glut3 was upregulated, suggesting that the neuronal cells are undersupplied with unchanged glucose import through the BBB. The current state of BBB research after antipsychotic drug medication is not very progressed, although BBB pathology is recognized as a crucial factor for many neurological disorders (Pollack et al., 2017). Chronic APD administration induces alterations in the BBB (Ben‐Shachar, Livne, Spanier, Leenders, & Youdim, 1994), and clozapine or haloperidol at therapeutic concentrations could strongly impair the bioenergetic state of human microvascular endothelial cells (Elmorsy & Smith, 2015). Chronic administration of clozapine seems to elicit hypoglycemia in rat hypothalamic cells, which is corrected by increased hepatic glucose output (HGO) (Smith, Chaussade, Vickers, Jensen, & Shepherd, 2008), arising in females from glycogenolysis (von Wilmsdorff et al., 2014). Wotanis, Hanak, Wettstein, and Black (2003) could show that both antipsychotics elicited fewer significant changes after chronic administration over a period of 20 days than acute application. Therefore, we conclude that rats with long‐term clozapine medication tend to stabilize glucose homeostasis in the hypothalamus to avoid the negative effects of chronic hypoglycemia, possibly induced by effects of APD medication on dopaminergic pathways, and to provide the necessary glucose supply for astrocytes and neuronal cells by sex‐dependent adaptation of glucose transporters and hepatic glucose release.

Clozapine and haloperidol do not only differ in dopamine receptor expression and glucose metabolism. Recently, we have found slightly elevated insulin serum levels in haloperidol‐medicated rats with decreased food intake in females, but no changes in clozapine‐treated groups, although fasting glucose was highly increased in females (von Wilmsdorff et al., 2013). Clozapine seems to directly inhibit insulin secretion by inhibition of Glut2 expression in rat pancreatic islet cells (Liu, Ji, Xiao, & Wang, 2010). Furthermore, in contrast to haloperidol, ghrelin levels are elevated in clozapine‐medicated rats (von Wilmsdorff et al., 2013), possibly modulating insulin secretion and thereby glucose metabolism, as seen in rat and human pancreatic islets (Broglio et al., 2002; Egido, Rodriguez‐Gallardo, Silvestre, & Marco, 2002). The orexigenic hormone ghrelin is secreted by gastric mucosa and pancreas, and its main site of action is the hypothalamic melanocortin system, consisting of the pro‐opiomelanocortin (POMC)‐expressing neurons, the neuropeptide Y (NPY), and agouti‐related peptide (AgRP)‐coexpressing neurons and neurons, expressing MC3R and MC4R (Kim, Leyva, & Diano, 2014). Ghrelin increases food intake and decreases fat oxidation, which chronically contribute to increased adiposity (Dos‐Santos, Reis, Perello, Ferguson, & Mecawi, 2019). In the hypothalamic arcuate nucleus of male SD rats, ghrelin activates the intracellular energy sensor AMPK in orexigenic NPY neurons, which leads to activation of these neurons (Kohno, Sone, Minokoshi, & Yada, 2008). Kirk, Cahir, and Reynolds (2006) found increased hypothalamic NPY expression under clozapine but not haloperidol in male SD rats. Furthermore, Yang, Qi, and Yang (2015) could show that stimulation of astrocytes in the arcuate nucleus reduces ghrelin‐evoked food intake by a mechanism of adenosine‐mediated inactivation of the orexigenic AgRP neurons via adenosine A_1_ receptors. We assume that under clozapine medication, increased body weight and fat tissue only in male SD rats without increased food intake (von Wilmsdorff et al., 2013) were possibly induced by interaction of ghrelin and the melanocortin system. Sex‐dependent differences were found for NPY in rat hypothalamus (Rugarn, Hammar, Theodorsson, Theodorsson, & Stenfors, 1999) and for MC4R in mouse brain (Qu et al., 2014) but not for ghrelin (Gayle, Desai, Casillas, Beloosesky, & Ross, 2006; von Wilmsdorff et al., 2013). In our study, hypothalamic MC4R protein expression did not differ significantly under APD medication with a trend to decreased receptors in medicated females (Figure 3), pointing to the deactivation of the melanocortin anorexigenic system. Further investigations are needed to explain the detailed mechanisms of sex‐dependent antipsychotic drug effects on the central melanocortin system including its regulation of peripheral insulin secretion via ghrelin in cooperation with pancreatic MC4R and autonomic neurons as seen in rats (Mansour et al., 2010).

This study provides further information on the metabolic effects of haloperidol and clozapine in hypothalamic tissue but has several limitations, and the results can only be transferred to human physiology with caution. However, animal studies permit the examination of medication effects separate from the disease process, but these effects might differ in the presence of the respective disease, for example in schizophrenia (Andreazza et al., 2015). Prepubertal social isolation is known to increase stress reactivity in male Sprague Dawley rats (Vargas, Junco, Gomez, & Lajud, 2016). In adult male Wistar rats, chronic isolation compromises the HPA axis and leads to a higher vulnerability of specific hippocampal interneurons (Filipovic, Zlatkovic, Gass, & Inta, 2013). Turner, Sunohara‐Neilson, Ovari, Healy, and Leri (2014) found that male Sprague Dawley rats, irrespective of single or pair housing, develop reduced HPA axis activity over time under standard laboratory housing conditions. In addition, rats did not differ in body weight and food consumption under social isolation. In our study, the rats were separated after puberty and the results of the antipsychotic drug‐treated groups were compared with the respective control group; therefore, we consider the effect of postpubertal individual housing in adult rats to be low. We noted a high variability of side effects under antipsychotic medication among Sprague Dawley rats. Admittedly, in our explorative study, the sample size is low and further investigations with a higher number of animals per group are needed in order to ensure our results.

## 5 CONCLUSION

The atypical antipsychotic clozapine acts differently to the typical haloperidol on hypothalamic glucose metabolism. Hypothalamic neurons seem to be undersupplied with glucose in clozapine‐medicated rats, probably caused by higher local glucose demand or inhibition of glucose uptake by clozapine. The increased glucose consumption with coincident increase of lactate levels in clozapine‐medicated rats points to enhanced glycolysis and is possibly linked to sex‐specific D2R activation or deactivation. The high hypothalamic glucose consumption is probably saturated by hepatic glycogenolysis in females and/or gluconeogenesis in males.

## CONFLICT OF INTEREST

The authors declare no conflict of interest.

## AUTHOR CONTRIBUTION

Marie‐Luise Bouvier participated in data collection, analyzed the data, and wrote this manuscript. Karin Fehsel supported the methodological part with helpful discussions and revised the manuscript. Andrea Schmitt, Eva Meisenzahl‐Lechner and Wolfgang Gaebel revised the manuscript. Martina von Wilmsdorff engaged in study conception and data collection and revised the manuscript.

## Data Availability

The data that support the findings of this study are available from the corresponding author upon reasonable request.
